# Inverse Relationship Between Serum Carotenoid Levels and Cardiovascular‐Kidney‐Metabolic Syndrome Among the General Adult Population

**DOI:** 10.1111/1753-0407.70046

**Published:** 2025-01-30

**Authors:** Mengli Chen, Shuyue Cai, Qinfeng Jia, Yifang Suo, Yuan Tang, Yanping Shi, Xu Zhu, Haifeng Zhang

**Affiliations:** ^1^ Department of Cardiology The Affiliated Suzhou Hospital of Nanjing Medical University, Suzhou Municipal Hospital, Gusu School, Nanjing Medical University Suzhou China; ^2^ State Key Laboratory for Innovation and Transformation of Luobing Theory, Department of Cardiology The First Affiliated Hospital of Nanjing Medical University, Jiangsu Province Hospital Nanjing China

**Keywords:** cardiovascular disease, carotenoids, kidney disease, metabolic syndrome, NHANES, WQS

## Abstract

**Objective:**

To examine the relationship between serum carotenoid levels and cardiovascular‐kidney‐metabolic (CKM) syndrome in a representative sample of US adults.

**Methods:**

Data from the fasting subsample of the NHANES 2017–2018 were analyzed using a survey‐weighted approach to ensure the findings are representative of the broader US adult population. Serum levels of α‐carotene, β‐carotene, β‐cryptoxanthin, lutein/zeaxanthin, and lycopene were measured using high‐performance liquid chromatography. CKM syndrome stages were defined according to the 2023 American Heart Association guidelines, with advanced CKM syndrome categorized as stages 3 or 4. Associations between serum carotenoids and advanced CKM syndrome were assessed using logistic regression and weighted quantile sum (WQS) regression.

**Results:**

The study included 1671 adults aged 20 years and older, with a mean age of 48.7 years and a gender distribution of 50.9% female and 49.1% male. Higher serum levels of α‐carotene, β‐carotene, α‐cryptoxanthin, lutein/zeaxanthin, and lycopene were inversely associated with advanced CKM syndrome. Specifically, compared to the lowest quartile, the highest quartile of α‐carotene had an odds ratio (OR) of 0.29 (95% CI: 0.16–0.55), β‐carotene 0.35 (95% CI: 0.16–0.78), α‐cryptoxanthin 0.23 (95% CI: 0.11–0.49), lutein/zeaxanthin 0.26 (95% CI: 0.14–0.48), and lycopene 0.58 (95% CI: 0.35–0.98). However, β‐cryptoxanthin did not show a significant association. Moreover, the combined effect of all carotenoids was significantly negatively correlated with advanced CKM syndrome (OR = 0.67, 95% CI: 0.53–0.86), with lutein/zeaxanthin contributing the most (44.56%).

**Conclusions:**

Elevated serum carotenoid levels are inversely associated with the prevalence of advanced CKM syndrome in a dose‐dependent manner, with this association remaining consistent across diverse demographic and health subgroups.

**Summary**

- This cross‐sectional study found that higher serum levels of α‐carotene, β‐carotene, lutein/zeaxanthin, and lycopene were significantly associated with a lower prevalence of advanced cardiovascular‐kidney‐metabolic (CKM) syndrome.
- The combined effect of six carotenoids showed an inverse relationship, with lutein/zeaxanthin contributing the most.
- These findings suggest a potential protective association between carotenoid levels and advanced CKM outcomes in US adults.

## 1 Introduction

In October 2023, the American Heart Association (AHA) introduced the concept of cardiovascular‐kidney‐metabolic (CKM) syndrome, highlighting the interrelated nature of metabolic disorders—such as diabetes and obesity—alongside kidney diseases and cardiovascular conditions [1]. CKM syndrome is characterized as a systemic disorder marked by intricate interactions among metabolic risk factors, chronic kidney disease (CKD), and cardiovascular diseases [1]. This concept is increasingly supported by research [2], which shows a consistent association between metabolic abnormalities, cardiovascular disease (CVD), and CKD, with these conditions frequently occurring together in patients [3]. Moreover, each of these diseases is linked to poor survival rates and elevated mortality [3]. The shared underlying mechanisms among these conditions suggest that the progression of one may exacerbate the others, potentially leading to worse outcomes for patients.

Beyond its clinical framework, the CKM concept underscores the importance of addressing Social Determinants of Health (SDOH) to achieve equitable prevention and management [1]. According to the AHA, systematic integration of SDOH—such as financial strain, access to healthcare, education, and environmental influences—into CKM care models is essential for mitigating health disparities and improving outcomes [1]. The overarching goal of the CKM framework is to guide interdisciplinary care that incorporates both clinical and social factors, reflecting the multifaceted nature of CKM syndrome [1]. However, while the present study does not directly address SDOH or the broader CKM framework, it aligns with these objectives by examining potential modifiable factors—specifically serum carotenoids and dietary carotenoid intake—that may contribute to CKM prevention and management. Understanding these relationships can inform strategies to complement SDOH‐focused interventions within the CKM framework.

Carotenoids are potent antioxidants that protect cells from oxidative damage by neutralizing free radicals [4]. Of the more than 700 identified carotenoids, six—α‐carotene, β‐carotene, β‐cryptoxanthin, lycopene, lutein, and zeaxanthin—account for over 95% of those present in human circulation [5]. Epidemiological evidence indicates that elevated serum carotenoid levels are associated with a reduced risk of cardiovascular diseases, diabetes, kidney diseases, and other metabolic disorders [6, 7]. Furthermore, several cross‐sectional studies have demonstrated that higher serum concentrations of carotenoids correspond with a lower prevalence of metabolic syndrome [8, 9].

Given their antioxidant properties, carotenoids have been extensively studied for their potential impact on CVD, metabolic disorders, and CKD. Numerous observational studies have demonstrated an inverse relationship between carotenoid levels and the risk of atherosclerotic cardiovascular disease [10]. In a study involving 3107 adults with diabetes, elevated serum β‐carotene levels were associated with a reduced risk of cardiovascular mortality over a 14‐year follow‐up period [7]. Additionally, Hirahatake et al. conducted a prospective cohort analysis examining the link between serum carotenoids and kidney function, finding that higher carotenoid levels significantly reduced the risk of rapid kidney function decline over 5 years [11]. These findings suggest that elevated serum carotenoid levels may confer protective benefits across various health conditions, including CKM syndrome.

The mixed findings from these studies indicate that while carotenoids may offer some protective effects against CKM syndrome, the relationship is not consistently observed across all research. Some studies have shown that higher serum carotenoid levels are associated with a reduced risk of developing CVD [12], whereas others have found no significant association between serum carotenoid levels and CVD risk [13]. For example, a recent study reported that increased dietary carotenoid intake was linked to a lower prevalence of CKD among females, but no significant association was found for males [14]. Additionally, higher dietary intake of carotenoids, particularly β‐carotene, has been associated with a reduced risk of developing type 2 diabetes (T2D) [15]. However, a randomized controlled trial found no evidence that β‐carotene supplementation alone protects against metabolic syndrome [16]. Notably, no prior study has specifically examined the correlation between dietary carotenoid intake and CKM syndrome. Further research is necessary to clarify the potential benefits of carotenoids and to establish clear guidelines for their supplementation in the prevention and management of CKM syndrome.

No previous research has examined the association between serum carotenoid levels and the prevalence of CKM syndrome. This study hypothesizes that higher serum carotenoid levels and dietary carotenoid intake are associated with a lower prevalence of CKM syndrome. Utilizing data from the National Health and Nutrition Examination Survey (NHANES), this research aims to investigate these associations and provide valuable insights into the potential role of carotenoids in CKM syndrome.

## 2 Materials and Methods

### 2.1 Study Population

The NHANES, conducted by the National Center for Health Statistics (NCHS) under the Centers for Disease Control and Prevention (CDC), is a cross‐sectional study that evaluates the health and nutritional status of the US population. The survey combines interviews with physical examinations and laboratory tests, offering a comprehensive dataset that reflects the health of the US population. NHANES employs a complex, multistage probability sampling design to ensure that the results are nationally representative. The NCHS Research Ethics Review Board approved the research protocols, and all participants provided written informed consent. Data are publicly available from the NHANES website at https://www.cdc.gov/nchs/nhanes/index.htm.

The study initially involved 3036 participants from the fasting subsample of NHANES 2017–2018. After excluding 325 individuals due to missing laboratory results or insufficient fasting duration (8 to < 24 h), 2711 participants remained. Further exclusions were made for those under 20 years old (*n* = 416), missing serum carotenoid data (*n* = 613), and pregnant individuals (*n* = 11). The final analysis included 1671 participants.

### 2.2 Measurement of Serum Carotenoid Levels

Carotenoids, including α‐carotene, β‐carotene, β‐cryptoxanthin, lutein/zeaxanthin, and lycopene, make up over 95% of the carotenoids in human plasma or serum [17]. The NHANES database measured serum concentrations of these carotenoids using a modified high‐performance liquid chromatography method with photodiode array detection. The specific carotenoids measured were α‐carotene, trans‐β‐carotene, cis‐β‐carotene, α‐cryptoxanthin, β‐cryptoxanthin, combined lutein/zeaxanthin, and total lycopene. Serum β‐carotene was determined by summing trans‐β‐carotene and cis‐β‐carotene concentrations. Detailed laboratory procedures and quality control protocols are available at https://wwwn.cdc.gov/Nchs/Nhanes/2017‐2018/VITAEC_J.htm.

### 2.3 Definition of CKM Syndrome

The stages of CKM Syndrome, ranging from 0 to 4, are defined based on the 2023 AHA Presidential Advisory on CKM Health [1]. The classification of stages is as follows: Stage 0: individuals in this stage are characterized by the absence of CKM risk factors, such as hypertension; Stage 1: this stage includes individuals with overweight, obesity, or dysfunctional adipose tissue, but without the presence of additional metabolic risk factors or CKD; Stage 2: participants in this stage have developed additional metabolic risk factors or present with moderate‐ to high‐risk CKD; Stage 3: individuals in this stage have very high‐risk CKD or are predicted to have a high 10‐year CVD risk; Stage 4: this final stage is characterized by the presence of established CVD, such as coronary artery disease. Detailed descriptions of these stages, as adapted to the NHANES data, are provided in Supporting Information. For the purpose of this study, advanced CKM syndrome was defined as stages 3 or 4, as these stages encompass individuals who either have or are at high risk of developing CVD.

### 2.4 Assessment of Covariates

In this analysis, several covariates were included to address potential confounding factors and enhance the validity of the findings. The demographic variables comprised age (continuous), sex (male or female), race/ethnicity (Non‐Hispanic White, Non‐Hispanic Black, or other), and education level (less than high school, high school, or more than high school). Socioeconomic status was assessed using the family poverty income ratio (PIR), categorized as ≤ 1.0, 1.1–3.0, or > 3.0, which represents the ratio of family income to a poverty threshold specific to family size, as defined by the US Department of Health and Human Services [18]. Drinking Status was categorized into nondrinker, low‐to‐moderate drinker (less than 2 drinks/day for men and less than 1 drink/day for women), and heavy drinker (2 or more drinks/day for men and 1 or more drinks/day for women) [7]. Physical Activity was classified as inactive (no leisure‐time physical activity), insufficiently active (moderate activity 1–5 times per week with MET of 3–6, or vigorous activity 1–3 times per week with MET > 6), and active (more frequent moderate or vigorous leisure‐time activity) [19]. The Healthy Eating Index (HEI), calculated from 24‐h dietary recall data, evaluates diet quality according to the 2015–2020 Dietary Guidelines for Americans [20]. The HEI comprises 13 components with a maximum score of 100, providing a detailed measure of adherence to key dietary recommendations.

### 2.5 Statistical Analyses

To address the complex multilevel sampling design of NHANES, all analyses incorporated sampling weights, strata, and primary sampling units (PSUs) to ensure nationally representative estimates. Continuous variables were reported as mean ± standard deviation for normally distributed data and as median with quartiles for nonnormally distributed data. Normal distributions were analyzed using Student's *t*‐test, while nonnormal distributions were assessed with the Mann–Whitney *U* test. Chi‐square tests were used for categorical variables. The Benjamini–Hochberg (BH) method was used to control the false discovery rate (FDR) for multiple comparisons.

The analysis focused on adults aged 20 years and older from the NHANES fasting subsample, which represents approximately half of the US noninstitutionalized population. Missing data rates for physical examination and laboratory measurements in this subsample were low (< 10%). Missing values for covariates were handled using multiple imputation with the “mice” package, employing a random forest algorithm.

The effect of individual serum carotenoids (α‐carotene, β‐carotene, α‐cryptoxanthin, β‐cryptoxanthin, combined lutein/zeaxanthin, and total lycopene) on advanced CKM syndrome (Stages 3 or 4) was evaluated using survey‐weighted logistic regression models. Results are presented as odds ratios (ORs) with 95% confidence intervals (CIs). Variance inflation factors (VIFs) were calculated to assess multicollinearity among covariates in the regression models, and no evidence of multicollinearity was identified. Restricted cubic splines (RCS) with three knots placed at the 10th, 50th, and 90th percentiles of the independent variable distribution were used to evaluate the linearity assumption between the independent variables and the odds of the outcome, and to explore potential nonlinear relationships.

Spearman's correlation coefficients were calculated to assess correlations among the serum carotenoids. The combined effect of the six carotenoids on advanced CKM syndrome was analyzed using weighted quantile sum (WQS) regression. This approach involved creating a WQS index by assigning weights to each carotenoid concentration via bootstrap sampling. Carotenoid concentrations were divided into deciles, and the data were split into training (40%) and validation (60%) sets, with 5000 bootstrap iterations. The combined effect of the six carotenoids was assessed by regressing the WQS index on advanced CKM syndrome using a fully adjusted model. Data analysis was conducted using R (version 4.1.2), the “survey” package was used for the weight analysis, and the “gWQS” package was employed for WQS regression. Statistical significance was defined as a *p* value less than 0.05.

## 3 Results

### 3.1 Characteristics of Study Participants

The study analyzed 1671 adults aged 20 years and older from the NHANES 2017–2018 fasting subsample, identifying 391 participants (23.4%) with advanced CKM syndrome (Stages 3 or 4) (Table 1). The mean age was 48.70 years, with the advanced CKM group significantly older (68.45 vs. 44.55 years, *p* < 0.01). Gender distribution was balanced (50.89% female, 49.11% male), with a higher prevalence of males in the advanced CKM group (54.66%) and a slight female majority in the non‐CKM group (52.06%), though this difference was not statistically significant (*p* = 0.08). Significant differences were observed in race/ethnicity (*p* = 0.04), with Non‐Hispanic Whites more common in the advanced CKM group, and in education levels, with lower education more prevalent in the advanced CKM group (*p* = 0.02). Family PIR differed, with more individuals in the middle PIR category (1.1–3.0) in the advanced CKM group (*p* = 0.04). There were no significant differences in serum cotinine levels. However, lifestyle factors, such as drinking status and physical activity, showed marked differences, with more nondrinkers and inactive individuals in the advanced CKM group (*p* < 0.01). Healthy Eating Index scores were consistent across both groups (*p* = 0.90).

**TABLE 1:** Survey‐weighted characteristics of adults ≥ 20 years in the fasting subsample of NHANES 2017–2018.

Characteristics	Overall (*n* = 1671)	Advanced CKM syndrome (Stages 3 or 4)		*p* *
		No (*n* = 1280)	Yes (*n* = 391)	
Age, years	48.70 ± 0.87	44.55 ± 0.94	68.45 ± 0.69	< 0.01
Sex, %				0.08
Female	872 (50.89)	697 (52.06)	175 (45.34)	
Male	799 (49.11)	583 (47.94)	216 (54.66)	
Race/ethnicity, %				0.04
Non‐Hispanic White	558 (62.07)	378 (60.72)	180 (68.49)	
Non‐Hispanic Black	397 (11.93)	300 (11.77)	97 (12.67)	
Other race	716 (26.00)	602 (27.51)	114 (18.85)	
Education level, %				0.02
Below high school	349 (12.12)	245 (10.72)	104 (18.82)	
High school	391 (28.64)	289 (28.13)	102 (31.07)	
Above high school	931 (59.24)	746 (61.15)	185 (50.11)	
Family PIR, %				0.04
≤ 1.0	301 (11.85)	233 (11.69)	68 (12.60)	
1.1–3.0	744 (39.18)	546 (37.15)	198 (48.83)	
> 3.0	626 (48.97)	501 (51.16)	125 (38.57)	
Serum cotinine, ng/mL				0.44
< 0.05	937 (57.69)	704 (56.94)	233 (61.26)	
0.05–2.99	331 (17.47)	252 (17.20)	79 (18.78)	
> 2.99	403 (24.84)	324 (25.87)	79 (19.96)	
Drinking status, %				< 0.01
Nondrinker	523 (22.75)	344 (19.25)	179 (39.39)	
Low‐to‐moderate drinker	1004 (66.49)	815 (68.66)	189 (56.18)	
Heavy drinker	144 (10.75)	121 (12.08)	23 (4.43)	
Physical activity, %				< 0.01
Inactive	437 (20.99)	277 (17.89)	160 (35.72)	
Insufficiently active	485 (29.18)	373 (29.01)	112 (30.01)	
Active	749 (49.83)	630 (53.11)	119 (34.28)	
Healthy eating index	49.20 ± 0.75	49.23 ± 0.77	49.05 ± 1.36	0.90

*Note:* Normally distributed continuous variables are described as means ± SEs. Sampling weights were applied for calculation of demographic descriptive statistics; *N* reflect the study sample while percentages reflect the survey‐weighted data.

Abbreviations: CKM, cardiovascular‐kidney‐metabolic; PIR, poverty income ratio.

* *p* values were adjusted for multiple comparisons using the Benjamini–Hochberg method to control the false discovery rate.

The distribution of serum and dietary carotenoids in adults in the NHANES 2017–2018 is shown in Table S1. Median serum concentrations were as follows: α‐carotene at 2.81 μg/dL (IQR: 1.50–5.51 μg/dL), β‐carotene at 13.60 μg/dL (IQR: 7.92–23.11 μg/dL), α‐cryptoxanthin at 2.28 μg/dL (IQR: 1.63–3.22 μg/dL), β‐cryptoxanthin at 6.31 μg/dL (IQR: 3.90–10.50 μg/dL), lutein/zeaxanthin at 16.10 μg/dL (IQR: 11.00–23.30 μg/dL), and lycopene at 37.50 μg/dL (IQR: 25.80–50.40 μg/dL). Dietary intake levels showed even broader variation: α‐carotene at 45.00 μg/day (IQR: 11.00–186.00 μg/day), β‐carotene at 797.00 μg/day (IQR: 327.00–2607.00 μg/day), β‐cryptoxanthin at 26.00 μg/day (IQR: 7.00–80.00 μg/day), lycopene at 782.00 μg/day (IQR: 350.00–1551.00 μg/day), and lutein/zeaxanthin at 1756.00 μg/day (IQR: 1.00–6213.00 μg/day).

In addition, Table S2 presents the survey‐weighted characteristics of adults across CKM stages 0–4. The analysis reveals significant reductions in serum carotenoid levels—α‐carotene, β‐carotene, α‐cryptoxanthin, β‐cryptoxanthin, lutein/zeaxanthin, and lycopene—as CKM syndrome advances. Median levels of all six carotenoids were highest in Stage 0 and decreased progressively through Stages 1 to 4.

### 3.2 Associations Between Serum Carotenoids and Advanced CKM Syndrome

**FIGURE 1:**
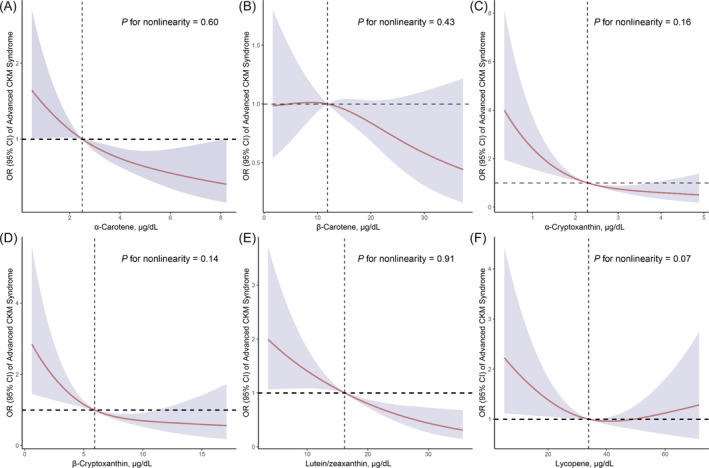
Restricted cubic spline (RCS) analysis with multivariate‐adjusted associations of serum carotenoids levels with advanced CKM syndrome (Stages 3 or 4) among adults in NHANES 2017–2018. Models are adjusted for age (continuous), sex (male or female), race (Non‐Hispanic White, Non‐Hispanic Black, or other), education level (below high school, high school, or above high school), family PIR (≤ 1.0, 1.1–3.0, or > 3.0), serum cotinine (< 0.05, 0.05–2.99, or > 2.99), drinking status (nondrinker, low‐to‐moderate drinker, heavy drinker), physical activity (inactive, insufficiently active, or active), and HEI (continuous).

The multiple logistic regression analysis revealed significant associations between serum carotenoid levels and advanced CKM syndrome among adults (Table 2). In both the crude model and Model, all carotenoids except β‐carotene were inversely associated with advanced CKM syndrome. After adjusting for multivariable factors, significant inverse associations persisted for α‐carotene, β‐carotene, α‐cryptoxanthin, lutein/zeaxanthin, and lycopene. Specifically, compared to the lowest quartile, the highest quartile of each carotenoid was associated with reduced odds of advanced CKM syndrome: α‐carotene (OR = 0.29 [95% CI: 0.16–0.55], *p*_trend_ < 0.01), β‐carotene (OR = 0.35 [0.16–0.78], *p*_trend_ = 0.05), α‐cryptoxanthin (OR = 0.23 [0.11–0.49], *p*_trend_ < 0.01), lutein/zeaxanthin (OR = 0.26 [0.14–0.48], *p*_trend_ < 0.01), and lycopene (OR = 0.58 [0.35–0.98], *p*_trend_ = 0.05). In contrast, β‐cryptoxanthin did not show a significant association after multivariable adjustment. Additionally, RCS regression indicated a linear relationship between all serum carotenoids levels and advanced CKM syndrome (Figure 1).

**TABLE 2:** Associations of quartiles of serum carotenoids levels with the prevalence of advanced CKM syndrome (Stages 3 or 4) among adults in NHANES 2017–2018.

	Serum carotenoids (μg/dL)				
	Quartile 1	Quartile 2	Quartile 3	Quartile 4	*p*_trend_ *
α‐Carotene					
Crude	1 [Reference]	0.88 (0.50–1.56)	0.90 (0.54–1.49)	0.56 (0.37–0.85)	0.04
Model 1	1 [Reference]	0.59 (0.27–1.28)	0.48 (0.23–0.98)	0.26 (0.15–0.45)	< 0.01
Model 2	1 [Reference]	0.63 (0.35–1.15)	0.54 (0.27–1.10)	0.29 (0.16–0.55)	< 0.01
β‐Carotene					
Crude	1 [Reference]	0.88 (0.39–1.95)	1.09 (0.62–1.90)	0.74 (0.41–1.37)	0.55
Model 1	1 [Reference]	0.75 (0.24–2.33)	0.77 (0.34–1.76)	0.30 (0.15–0.61)	0.02
Model 2	1 [Reference]	0.85 (0.31–2.33)	0.87 (0.39–1.97)	0.35 (0.16–0.78)	0.05
α‐Cryptoxanthin					
Crude	1 [Reference]	0.56 (0.35–0.88)	0.34 (0.22–0.53)	0.21 (0.12–0.39)	< 0.01
Model 1	1 [Reference]	0.56 (0.36–0.85)	0.35 (0.20–0.64)	0.22 (0.11–0.45)	< 0.01
Model 2	1 [Reference]	0.62 (0.38–1.01)	0.38 (0.21–0.66)	0.23 (0.11–0.49)	< 0.01
β‐Cryptoxanthin					
Crude	1 [Reference]	0.50 (0.35–0.71)	0.27 (0.15–0.47)	0.37 (0.19–0.73)	< 0.01
Model 1	1 [Reference]	0.63 (0.37–1.06)	0.39 (0.20–0.75)	0.42 (0.20–0.88)	0.02
Model 2	1 [Reference]	0.59 (0.36–0.96)	0.44 (0.21–0.90)	0.50 (0.22–1.14)	0.06
Lutein/zeaxanthin					
Crude	1 [Reference]	0.73 (0.44–1.21)	1.01 (0.63–1.63)	0.53 (0.37–0.76)	0.04
Model 1	1 [Reference]	0.47 (0.27–0.84)	0.64 (0.39–1.06)	0.23 (0.12–0.41)	< 0.01
Model 2	1 [Reference]	0.50 (0.27–0.94)	0.74 (0.49–1.13)	0.26 (0.14–0.48)	< 0.01
Lycopene					
Crude	1 [Reference]	0.57 (0.35–0.92)	0.34 (0.22–0.51)	0.29 (0.18–0.46)	< 0.01
Model 1	1 [Reference]	0.62 (0.33–1.17)	0.63 (0.32–1.22)	0.51 (0.29–0.89)	0.04
Model 2	1 [Reference]	0.68 (0.36–1.28)	0.69 (0.39–1.21)	0.58 (0.35–0.98)	0.05

*Note:* Data are presented as OR (95% CI) unless indicated otherwise; Model 1 was adjusted for age (continuous), sex (male or female), and race (Non‐Hispanic White, Non‐Hispanic Black, or Other); Model 2 was adjusted for Model 1 plus education level (below high school, high school, or above high school), family PIR (≤ 1.0, 1.1–3.0, or > 3.0), serum cotinine (< 0.05, 0.05–2.99, or > 2.99), drinking status (nondrinker, low‐to‐moderate drinker, heavy drinker), physical activity (inactive, insufficiently active, or active), and Healthy Eating Index (continuous).

* *p* values were adjusted for multiple comparisons using the Benjamini–Hochberg method to control the false discovery rate.

### 3.3 Associations Between the Combine of Six Serum Carotenoids and Advanced CKM Syndrome

**FIGURE 2:**
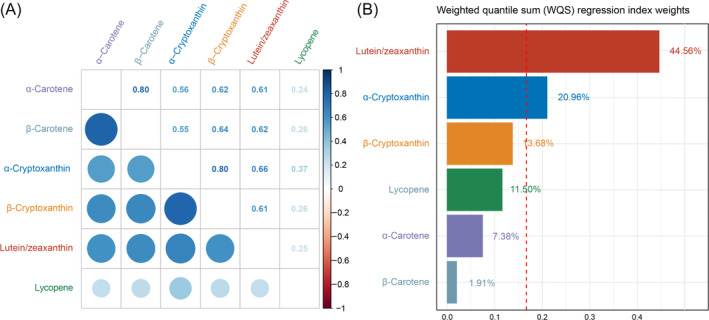
Pairwise Spearman correlation coefficients among serum carotenoids (A). WQS model regression index weights for each carotenoid for advanced CKM syndrome (Stages 3 or 4) among adults in NHANES 2017–2018 (B). WQS regression was adjusted as age (continuous), sex (male or female), race (Non‐Hispanic White, Non‐Hispanic Black, or other), education level (below high school, high school, or above high school), family PIR (≤ 1.0, 1.1–3.0, or > 3.0), serum cotinine (< 0.05, 0.05–2.99, or > 2.99), drinking status (nondrinker, low‐to‐moderate drinker, heavy drinker), physical activity (inactive, insufficiently active, or active), and HEI (continuous). WQS: weighted quantile sum.

The pairwise Spearman's correlation coefficients among the six serum carotenoids ranged from 0.25 to 0.80, indicating varying degrees of correlation (Figure 2A). The WQS regression analysis demonstrated a significant negative association between the combined levels of all six carotenoids (α‐carotene, β‐carotene, α‐cryptoxanthin, β‐cryptoxanthin, lutein/zeaxanthin, and lycopene) and advanced CKM syndrome, with an OR of 0.67 (95% CI: 0.53–0.86, *p* < 0.01). Figure 2B presents the estimated weights for each carotenoid's contribution to the overall association with advanced CKM syndrome. Notably, lutein/zeaxanthin had the highest weight, contributing 44.56% to the combined effect, suggesting a strong association with lower prevalence of advanced CKM syndrome.

### 3.4 Subgroup Analysis and Sensitivity Analysis

The subgroup analyses examining the associations between serum lutein/zeaxanthin levels and advanced CKM syndrome revealed that none of the interaction terms were statistically significant (Table 3). This indicates that the association between serum lutein/zeaxanthin levels and advanced CKM syndrome was consistent across subgroups defined by these demographic and behavioral factors (including age, sex, race/ethnicity, education level, family PIR, serum cotinine, drinking status, and physical activity), with no notable differences observed.

**TABLE 3:** Stratified analyses of the associations between quartiles of serum lutein/zeaxanthin levels with the prevalence of advanced CKM syndrome (Stages 3 or 4) among adults in NHANES 2017–2018.

Subgroups	*N*	Serum lutein/zeaxanthin levels				*p*‐int*
		Quartile 1	Quartile 2	Quartile 3	Quartile 4	
Age						0.33
20–59	1032	1 [Reference]	0.33 (0.11–1.03)	0.87 (0.35–2.13)	0.47 (0.17–1.35)	
≥ 60	639	1 [Reference]	0.80 (0.41–1.57)	1.04 (0.47–2.32)	0.41 (0.19–0.86)	
Sex						0.29
Female	872	1 [Reference]	0.27 (0.13–0.59)	0.62 (0.27–1.43)	0.17 (0.07–0.38)	
Male	799	1 [Reference]	0.90 (0.44–1.82)	1.25 (0.56–2.79)	0.45 (0.20–1.01)	
Race/ethnicity						0.38
Non‐Hispanic White	558	1 [Reference]	0.54 (0.20–1.49)	0.86 (0.41–1.78)	0.28 (0.10–0.76)	
Non‐Hispanic Black	397	1 [Reference]	0.50 (0.20–1.30)	1.08 (0.31–3.83)	0.25 (0.07–0.88)	
Other	716	1 [Reference]	0.24 (0.13–0.42)	0.28 (0.16–0.49)	0.12 (0.07–0.23)	
Education level						0.41
Below high school	349	1 [Reference]	0.15 (0.03–0.78)	0.28 (0.09–0.86)	0.22 (0.05–0.97)	
High school	391	1 [Reference]	0.32 (0.09–1.09)	1.00 (0.25–3.91)	0.23 (0.07–0.83)	
Above high school	931	1 [Reference]	0.79 (0.31–2.02)	0.68 (0.25–1.86)	0.24 (0.10–0.58)	
Family PIR						0.30
≤ 1.0	301	1 [Reference]	0.15 (0.05–0.40)	0.55 (0.20–1.50)	0.41 (0.10–1.72)	
1.1–3.0	744	1 [Reference]	0.55 (0.22–1.35)	0.88 (0.31–2.53)	0.28 (0.10–0.78)	
> 3.0	626	1 [Reference]	0.58 (0.22–1.53)	0.80 (0.26–2.46)	0.21 (0.05–0.80)	
Serum cotinine, ng/mL						0.30
< 0.05	937	1 [Reference]	0.46 (0.23–0.93)	0.43 (0.22–0.83)	0.21 (0.08–0.52)	
0.05–2.99	331	1 [Reference]	0.94 (0.13–6.66)	3.18 (0.51–19.68)	0.25 (0.06–1.04)	
> 2.99	403	1 [Reference]	0.46 (0.16–1.31)	1.32 (0.33–5.37)	0.27 (0.07–1.11)	
Drinking status						0.21
Nondrinker	523	1 [Reference]	1.24 (0.47–3.31)	1.37 (0.61–3.06)	0.42 (0.16–1.11)	
Low‐to‐moderate drinker	1004	1 [Reference]	0.33 (0.12–0.88)	0.53 (0.26–1.08)	0.19 (0.08–0.48)	
Heavy drinker	144	1 [Reference]	0.10 (0.03–0.40)	0.77 (0.08–7.51)	0.10 (0.01–1.38)	
Physical activity						0.29
Inactive	437	1 [Reference]	0.44 (0.13–1.51)	0.57 (0.20–1.58)	0.39 (0.12–1.33)	
Insufficiently active	485	1 [Reference]	0.32 (0.10–1.03)	0.14 (0.03–0.68)	0.12 (0.03–0.41)	
Active	749	1 [Reference]	0.62 (0.15–2.58)	1.96 (0.91–4.21)	0.19 (0.10–0.35)	

*Note:* Data are presented as OR (95% CI) unless indicated otherwise; Analyses were adjusted for age (continuous), sex (male or female), race (Non‐Hispanic White, Non‐Hispanic Black, or other), education level (below high school, high school, or above high school), family PIR (≤ 1.0, 1.1–3.0, or > 3.0), serum cotinine (< 0.05, 0.05–2.99, or > 2.99), drinking status (nondrinker, low‐to‐moderate drinker, heavy drinker), physical activity (inactive, insufficiently active, or active), and HEI (continuous) when they were not the strata variables.

Abbreviations: CKM, cardiovascular‐kidney‐metabolic; *p*‐int, *p* for interaction; PIR, poverty income ratio.

* *p* values were adjusted for multiple comparisons using the Benjamini–Hochberg method to control the false discovery rate.

Additional sensitivity analyses were conducted to verify the robustness of the associations between serum carotenoids and advanced CKM syndrome. First, adjusting for dietary carotenoid intakes did not significantly affect the inverse associations observed (Table S3). Second, incorporating dietary carotenoid supplements into the model retained significant associations for most carotenoids (Table S4). Third, when adjusting for both dietary intakes and supplements, the significant inverse relationships between serum carotenoids and advanced CKM syndrome persisted (Table S5). Additionally, as shown in Table S6, higher oxidative balance scores (OBS) were significantly associated with a lower prevalence of advanced CKM syndrome (Stages 3 or 4) across all models. Furthermore, Figure S2 demonstrates that the combined effects of six serum carotenoids were significantly associated with lower prevalence of CVD (OR = 0.64 [0.48–0.84]; *p* < 0.01) and combined CVD and CKD (OR = 0.78 [0.63–0.96]; *p* = 0.02), but not CKD alone (OR = 0.75 [0.50–1.12]; *p* = 0.17). Among the six carotenoids, lutein/zeaxanthin consistently showed the greatest contribution to these associations. These results collectively support the reliability and consistency of the observed inverse associations across various sensitivity analyses.

Table S7 displays the associations between quartiles of dietary carotenoid intake and advanced CKM syndrome. After adjusting for multivariable factors, the results indicate that dietary intakes of α‐carotene, β‐carotene, α‐cryptoxanthin, β‐cryptoxanthin, lutein/zeaxanthin, and lycopene did not exhibit significant trends or associations with advanced CKM syndrome across the quartiles.

## 4 Discussion

In this large cross‐sectional study involving 1671 adults aged 20 years and older from the NHANES 2017–2018 dataset, we observed that higher concentrations of several carotenoids in the bloodstream, including α‐carotene, β‐carotene, α‐cryptoxanthin, lutein/zeaxanthin, and lycopene, were associated with a lower prevalence of CKM syndrome. However, no significant association was found between β‐cryptoxanthin levels and CKM syndrome. The overall combined effect of carotenoids showed an inverse relationship with advanced stages of CKM syndrome. Notably, among the carotenoids studied, lutein/zeaxanthin showed the strongest association, accounting for 44.56% of the total combined influence on advanced CKM syndrome.

As a newly recognized concept, CKM syndrome is a complex and multifaceted condition that profoundly affects overall health and quality of life. For individuals with CKM syndrome, lifestyle management is crucial for slowing disease progression and preventing cardiovascular complications. The intake of antioxidant vitamins is particularly important in this context [21]. Carotenoids, which are potent antioxidants, are naturally occurring pigments found in fruits, vegetables, and other plant‐based foods. Certain carotenoids, such as beta‐carotene, serve as precursors to vitamin A, an essential nutrient for human health.

Research has provided evidence suggesting that carotenoids may play a protective role in metabolic diseases, CVD and CKD. Previous studies have indicated that increasing β‐carotene intake or elevating its serum or plasma levels may help to reduce cardiovascular risk [22]. For instance, a population‐based study of middle‐aged men found that higher levels of β‐carotene in the bloodstream were associated with a reduced risk of developing congestive heart failure [23]. Additionally, a cross‐sectional study based on NHANES data observed a link between higher carotenoid intake and lower odds of non‐alcoholic fatty liver disease (NAFLD) [24]. Moreover, a community‐based cross‐sectional analysis of 2148 participants aged 50–75 years in China reported an inverse correlation between serum carotenoid levels and the prevalence of metabolic syndrome [25]. Similarly, Ford et al., in a study conducted between 1988 and 1994 involving 8808 adults, found that individuals with metabolic syndrome generally had lower carotenoid levels, with the exception of lycopene [26]. Hirahatake et al. conducted a comprehensive analysis on the long‐term effects of serum carotenoids on renal health. Their study found that individuals in the highest quartile of serum carotenoids had a significantly lower risk of experiencing a rapid decline in renal function compared to those in the lowest quartile [11]. In the context of Type 2 diabetes, high serum β‐carotene levels were associated with an increased risk of cardiovascular mortality, while other individual carotenoids did not show significant effects on cardiovascular outcomes [7]. Although research on the relationship between carotenoids and the development of CKD is limited, recent studies suggest that dietary carotenoid intake may improve prognosis and reduce mortality risk in CKD patients [27]. Consistent with previous research, our study also found an inverse relationship between serum carotenoid levels and advanced CKM syndrome, suggesting that carotenoids may play a protective role in the development of CKM syndrome.

CKM syndrome is closely linked with inflammation, with systemic inflammation and oxidative stress playing pivotal roles in its development and progression [3]. Carotenoids, such as β‐carotene, lutein, zeaxanthin, and lycopene, are well‐regarded for their antioxidant properties. These antioxidants help mitigate oxidative stress and inflammation, both of which contribute to the onset and progression of various metabolic diseases. Reactive oxygen species (ROS) can activate inflammatory pathways, resulting in chronic inflammation. The kidneys are especially vulnerable to oxidative stress, as ROS can damage the glomeruli and tubules, impairing renal function and leading to chronic kidney disease and other renal complications [28]. Furthermore, oxidative stress plays a significant role in the remodeling of the heart and blood vessels, potentially leading to conditions such as left ventricular hypertrophy and atherosclerosis [29, 30]. Carotenoids, by reducing oxidative stress, help minimize the cellular damage caused by these ROS [31]. Additionally, carotenoids support the functionality of immune cells, enabling them to respond effectively to infections and other threats. Smoking and obesity are linked to lower serum carotenoid concentrations, which may indicate increased oxidative stress and a higher need for antioxidants in these individuals [32, 33]. Research has shown that lycopene can reduce the production of inflammation‐related cytokines, potentially lowering the risk of metabolic disorders like obesity and insulin resistance [26]. Overall, carotenoids help mitigate oxidative damage by neutralizing free radicals and singlet oxygen, thereby reducing oxidative stress [34]. As a sensitivity analysis, we further incorporated the OBS into our investigation. OBS is a composite score reflecting the balance between pro‐oxidant and antioxidant factors [35, 36, 37, 38], including dietary components such as carotenoids. Our analysis revealed that OBS was inversely associated with advanced CKM syndrome, aligning with our findings on carotenoid concentrations. This further supports the association between oxidative stress and CKM syndrome and highlights the potentially important role that maintaining a balanced oxidative environment may play in the disease state.

Our study found that lutein and zeaxanthin exhibit the strongest protective effects against CKM syndrome among the carotenoids examined. These carotenoids have been shown to improve endothelial function, which is crucial for the health of blood vessels [39]. Longer telomeres are linked to a reduced risk of age‐related diseases [40]. By preserving telomere length, lutein and zeaxanthin may help to lower the risk or delay the onset of conditions such as stroke, diabetes, cardiovascular disease, and cancer [40]. Additionally, these carotenoids have been found to reduce markers of oxidative cardiovascular damage, such as the oxidative modification of low‐density lipoprotein cholesterol, which is closely associated with atherosclerosis development [41]. Therefore, lutein holds promise as a beneficial nutrient for CKM patients by mitigating chronic inflammation. Moreover, potential synergistic effects between different carotenoids, or between carotenoids and other nutrients, could further enhance their protective impact against CKM syndrome.

However, the impact of individual carotenoids on CKM syndrome protection may vary. Notably, β‐cryptoxanthin was an exception, as its levels did not show a significant effect on CKM syndrome. This discrepancy may suggest that β‐cryptoxanthin is metabolized or utilized differently from other carotenoids, or it could reflect unique dietary patterns associated with β‐cryptoxanthin‐rich foods. While β‐cryptoxanthin has been shown to reduce the production of pro‐inflammatory cytokines and mitigate inflammation in animal models [42], more clinical studies are needed to clarify its specific relationship with CKM syndrome.

While dietary carotenoids are generally linked to a reduced risk of cardiovascular disease, the impact of carotenoid supplements on CKM syndrome in this study was less clear. The relationship between dietary carotenoids and CKM syndrome in the general population is inconsistent. Some studies suggest that carotenoids have a protective effect against CVD, while others find no significant associations, particularly with high‐dose β‐carotene supplements [43]. In fact, some trials have reported an increased risk of adverse cardiovascular events among participants receiving β‐carotene supplements compared with those who did not [44, 45]. A recent meta‐analysis indicated that dietary β‐carotene does not significantly affect the risk of major cardiovascular diseases [46]. At lower concentrations, β‐carotene is a potent antioxidant that neutralizes free radicals and protects cells from oxidative damage [47, 48]. However, at high concentrations, β‐carotene may lose its antioxidant efficacy [47, 48]. Previous studies have shown that serum carotenoid levels are more strongly associated with health outcomes than dietary intake [24]. The variability in carotenoid absorption due to factors such as genetics, dietary fat intake, and the presence of other dietary components may also play a role [49]. The limited sample size in our study may have contributed to inconclusive results. Therefore, future research with larger sample sizes is needed to achieve more reliable and statistically significant findings.

Our study found that higher serum carotenoid levels were inversely associated with the prevalence of advanced CKM syndrome, while no such association was observed with dietary carotenoid intake. This suggests that blood levels of carotenoids may better reflect their bioavailability and protective effects in the body compared to dietary intake. The inverse relationship between serum lutein/zeaxanthin and CKM syndrome, in particular, suggests that carotenoid‐rich diets could be beneficial for populations at risk for CKM syndrome. While our study did not find a direct link between dietary carotenoids and CKM, the strong association with serum carotenoids implies that increasing carotenoid intake through diet could potentially raise blood levels and provide a protective effect. These findings have important practical implications. If future studies confirm that increasing carotenoid intake can improve serum levels and reduce CKM syndrome risk, dietary interventions could become an accessible and cost‐effective strategy for preventing or managing CKM syndrome in high‐risk populations. Such interventions may include promoting the consumption of carotenoid‐rich foods such as leafy greens, colorful fruits, and vegetables, or even considering supplementation in certain populations. Further longitudinal and interventional studies are needed to explore the potential benefits of dietary strategies or supplementation aimed at improving carotenoid status and reducing CKM syndrome risk.

Our study has several limitations. First, the cross‐sectional design of this study limits the ability to establish causality, making it unclear whether serum carotenoid levels are a consequence of CKM syndrome or a contributing factor. Longitudinal studies are needed to explore the temporal relationship between carotenoid levels and CKM syndrome. Additionally, randomized controlled trials could help confirm the potential protective role of carotenoids and provide stronger evidence for their use in CKM prevention and management strategies. Second, some data, especially regarding dietary intake and lifestyle factors, were self‐reported, which may introduce recall bias or misclassification. Third, the relatively small sample size may limit the visibility of associations between dietary carotenoid levels and CKM syndrome prevalence. Fourth, while we adjusted for major sociodemographic characteristics and lifestyle factors, there may still be unmeasured variables that could affect the relationship between carotenoid levels and CKM syndrome. Finally, the CKM syndrome staging in our study was adapted from the AHA proposal due to the limitations of the publicly available NHANES data. As not all AHA‐recommended biomarkers and diagnostic criteria were available, our findings should be interpreted with caution. Future studies are warranted to incorporate a more comprehensive assessment of CKM syndrome staging using the full spectrum of AHA criteria.

## 5 Conclusion

This cross‐sectional analysis of a representative sample of US adults demonstrated a significant inverse relationship between the combined of all six serum carotenoids (α‐carotene, β‐carotene, α‐cryptoxanthin, β‐cryptoxanthin, lutein/zeaxanthin, and lycopene) and advanced CKM syndrome. Among these, lutein/zeaxanthin showed the strongest protective effect. Further research, including prospective studies and clinical trials, is warranted to confirm these associations and explore the underlying mechanisms driving the protective effects of carotenoids on CKM syndrome.

## Conflicts of Interest

The authors declare no conflicts of interest.

## Supporting information


**Data S1.** Supporting Information.

## References

[1] C. E. Ndumele , I. J. Neeland , K. R. Tuttle , et al., “A Synopsis of the Evidence for the Science and Clinical Management of Cardiovascular‐Kidney‐Metabolic (CKM) Syndrome: A Scientific Statement From the American Heart Association,” Circulation 148, no. 20 (2023): 1636–1664. 37807920 10.1161/CIR.0000000000001186

[2] M. Marassi and G. P. Fadini , “The Cardio‐Renal‐Metabolic Connection: A Review of the Evidence,” Cardiovascular Diabetology 22, no. 1 (2023): 195. 37525273 10.1186/s12933-023-01937-x PMC10391899

[3] S. A. Sebastian , I. Padda , and G. Johal , “Cardiovascular‐Kidney‐Metabolic (CKM) Syndrome: A State‐of‐the‐Art Review,” Current Problems in Cardiology 49, no. 2 (2024): 102344. 38103820 10.1016/j.cpcardiol.2023.102344

[4] M. Rodriguez‐Concepcion , J. Avalos , M. L. Bonet , et al., “A Global Perspective on Carotenoids: Metabolism, Biotechnology, and Benefits for Nutrition and Health,” Progress in Lipid Research 70 (2018): 62–93. 29679619 10.1016/j.plipres.2018.04.004

[5] G. Maiani , M. J. Castón , G. Catasta , et al., “Carotenoids: Actual Knowledge on Food Sources, Intakes, Stability and Bioavailability and Their Protective Role in Humans,” Molecular Nutrition & Food Research 53, no. Suppl 2 (2009): S194–S218. 19035552 10.1002/mnfr.200800053

[6] F. J. Pashkow , D. G. Watumull , and C. L. Campbell , “Astaxanthin: A Novel Potential Treatment for Oxidative Stress and Inflammation in Cardiovascular Disease,” American Journal of Cardiology 101, no. 10a (2008): 58d–68d. 18474276 10.1016/j.amjcard.2008.02.010

[7] Z. Qiu , X. Chen , T. Geng , et al., “Associations of Serum Carotenoids With Risk of Cardiovascular Mortality Among Individuals With Type 2 Diabetes: Results From NHANES,” Diabetes Care 45, no. 6 (2022): 1453–1461. 35503926 10.2337/dc21-2371

[8] M. A. Beydoun , M. R. Shroff , X. Chen , H. A. Beydoun , Y. Wang , and A. B. Zonderman , “Serum Antioxidant Status Is Associated With Metabolic Syndrome Among U.S. Adults in Recent National Surveys,” Journal of Nutrition 141, no. 5 (2011): 903–913. 21451127 10.3945/jn.110.136580 PMC3077890

[9] M. A. Beydoun , J. A. Canas , H. A. Beydoun , X. Chen , M. R. Shroff , and A. B. Zonderman , “Serum Antioxidant Concentrations and Metabolic Syndrome Are Associated Among U.S. Adolescents in Recent National Surveys,” Journal of Nutrition 142, no. 9 (2012): 1693–1704. 22810988 10.3945/jn.112.160416 PMC3417831

[10] A. Obana , M. Nakamura , A. Miura , M. Nozue , S. Muto , and R. Asaoka , “Association Between Atherosclerotic Cardiovascular Disease Score and Skin Carotenoid Levels Estimated via Refraction Spectroscopy in the Japanese Population: A Cross‐Sectional Study,” Scientific Reports 14, no. 1 (2024): 12173. 38806551 10.1038/s41598-024-62772-y PMC11133310

[11] K. M. Hirahatake , D. R. Jacobs , M. D. Gross , et al., “The Association of Serum Carotenoids, Tocopherols, and Ascorbic Acid With Rapid Kidney Function Decline: The Coronary Artery Risk Development in Young Adults (CARDIA) Study,” Journal of Renal Nutrition 29, no. 1 (2019): 65–73. 30098859 10.1053/j.jrn.2018.05.008

[12] D. Aune , N. Keum , E. Giovannucci , et al., “Dietary Intake and Blood Concentrations of Antioxidants and the Risk of Cardiovascular Disease, Total Cancer, and All‐Cause Mortality: A Systematic Review and Dose‐Response Meta‐Analysis of Prospective Studies,” American Journal of Clinical Nutrition 108, no. 5 (2018): 1069–1091. 30475962 10.1093/ajcn/nqy097 PMC6250988

[13] A. Goyal , M. B. Terry , and A. B. Siegel , “Serum Antioxidant Nutrients, Vitamin A, and Mortality in U.S. Adults,” Cancer Epidemiology, Biomarkers and Prevention 22, no. 12 (2013): 2202–2211. 10.1158/1055-9965.EPI-13-0381 PMC4026170 23897583

[14] Y. Shi , Y. Xu , and W. Zhou , “Dietary Carotenoids Intake and Sex Differences in Relation to Chronic Kidney Disease a Cross‐Sectional Assessment in the NHANES Study,” BMC Public Health 24, no. 1 (2024): 293. 38267887 10.1186/s12889-024-17771-z PMC10809643

[15] Y. W. Jiang , Z. H. Sun , W. W. Tong , et al., “Dietary Intake and Circulating Concentrations of Carotenoids and Risk of Type 2 Diabetes: A Dose‐Response Meta‐Analysis of Prospective Observational Studies,” Advances in Nutrition 12, no. 5 (2021): 1723–1733. 33979433 10.1093/advances/nmab048 PMC8483954

[16] S. Czernichow , A. C. Vergnaud , P. Galan , et al., “Effects of Long‐Term Antioxidant Supplementation and Association of Serum Antioxidant Concentrations With Risk of Metabolic Syndrome in Adults,” American Journal of Clinical Nutrition 90, no. 2 (2009): 329–335. 19491388 10.3945/ajcn.2009.27635

[17] A. Milani , M. Basirnejad , S. Shahbazi , and A. Bolhassani , “Carotenoids: Biochemistry, Pharmacology and Treatment,” British Journal of Pharmacology 174, no. 11 (2017): 1290–1324. 27638711 10.1111/bph.13625 PMC5429337

[18] Services USDoHaH , “Poverty Guidelines, Research, and Measurement,” http://aspe.hhs.gov/POVERTY/index.shtml.

[19] S. Beddhu , B. C. Baird , J. Zitterkoph , J. Neilson , and T. Greene , “Physical Activity and Mortality in Chronic Kidney Disease (NHANES III),” Clinical Journal of the American Society of Nephrology 4, no. 12 (2009): 1901–1906. 19820134 10.2215/CJN.01970309 PMC2798872

[20] S. I. Kirkpatrick , J. Reedy , S. M. Krebs‐Smith , et al., “Applications of the Healthy Eating Index for Surveillance, Epidemiology, and Intervention Research: Considerations and Caveats,” Journal of the Academy of Nutrition and Dietetics 118, no. 9 (2018): 1603–1621. 30146072 10.1016/j.jand.2018.05.020 PMC6730554

[21] K. Kalantar‐Zadeh and D. Fouque , “Nutritional Management of Chronic Kidney Disease,” New England Journal of Medicine 377, no. 18 (2017): 1765–1776. 29091561 10.1056/NEJMra1700312

[22] S. Mummidi , V. S. Farook , L. Reddivari , et al., “Serum Carotenoids and Pediatric Metabolic Index Predict Insulin Sensitivity in Mexican American Children,” Scientific Reports 11, no. 1 (2021): 871. 33441626 10.1038/s41598-020-79387-8 PMC7806924

[23] J. Karppi , S. Kurl , T. H. Mäkikallio , K. Ronkainen , and J. A. Laukkanen , “Serum β‐Carotene Concentrations and the Risk of Congestive Heart Failure in Men: A Population‐Based Study,” International Journal of Cardiology 168, no. 3 (2013): 1841–1846. 23333366 10.1016/j.ijcard.2012.12.072

[24] K. Christensen , T. Lawler , and J. Mares , “Dietary Carotenoids and Non‐Alcoholic Fatty Liver Disease Among US Adults, NHANES 2003‐2014,” Nutrients 11, no. 5 (2019): 1101, 10.3390/nu11051101. PMC6566688 31108934

[25] J. Liu , W. Q. Shi , Y. Cao , et al., “Higher Serum Carotenoid Concentrations Associated With a Lower Prevalence of the Metabolic Syndrome in Middle‐Aged and Elderly Chinese Adults,” British Journal of Nutrition 112, no. 12 (2014): 2041–2048. 25345663 10.1017/S000711451400316X

[26] E. S. Ford , A. H. Mokdad , W. H. Giles , and D. W. Brown , “The Metabolic Syndrome and Antioxidant Concentrations: Findings From the Third National Health and Nutrition Examination Survey,” Diabetes 52, no. 9 (2003): 2346–2352. 12941775 10.2337/diabetes.52.9.2346

[27] Y. Hu , X. Cai , N. Zhang , et al., “Relation Between Dietary Carotenoid Intake, Serum Concentration, and Mortality Risk of CKD Patients Among US Adults: National Health and Nutrition Examination Survey 2001‐2014,” Frontiers in Medicine 9 (2022): 871767. 35872751 10.3389/fmed.2022.871767 PMC9304649

[28] K. Daenen , A. Andries , D. Mekahli , A. Van Schepdael , F. Jouret , and B. Bammens , “Oxidative Stress in Chronic Kidney Disease,” Pediatric Nephrology 34, no. 6 (2019): 975–991. 30105414 10.1007/s00467-018-4005-4

[29] N. Tran , T. Garcia , M. Aniqa , S. Ali , A. Ally , and S. M. Nauli , “Endothelial Nitric Oxide Synthase (eNOS) and the Cardiovascular System: In Physiology and in Disease States,” American Journal of Biomedical Science & Research 15, no. 2 (2022): 153–177. 35072089 PMC8774925

[30] P. Y. Zhang , X. Xu , and X. C. Li , “Cardiovascular Diseases: Oxidative Damage and Antioxidant Protection,” European Review for Medical and Pharmacological Sciences 18, no. 20 (2014): 3091–3096. 25392110

[31] S. Voutilainen , T. Nurmi , J. Mursu , and T. H. Rissanen , “Carotenoids and Cardiovascular Health 2,” American Journal of Clinical Nutrition 83, no. 6 (2006): 1265–1271. 16762935 10.1093/ajcn/83.6.1265

[32] N. Yao , S. Yan , Y. Guo , et al., “The Association Between Carotenoids and Subjects With Overweight or Obesity: A Systematic Review and Meta‐Analysis,” Food & Function 12, no. 11 (2021): 4768–4782. 33977977 10.1039/d1fo00004g

[33] H. E. Gabriel , Z. Liu , J. W. Crott , et al., “A Comparison of Carotenoids, Retinoids, and Tocopherols in the Serum and Buccal Mucosa of Chronic Cigarette Smokers Versus Nonsmokers,” Cancer Epidemiology, Biomarkers & Prevention 15, no. 5 (2006): 993–999. 10.1158/1055-9965.EPI-05-0664 16702382

[34] J. Pirayesh Islamian and H. Mehrali , “Lycopene as a Carotenoid Provides Radioprotectant and Antioxidant Effects by Quenching Radiation‐Induced Free Radical Singlet Oxygen: An Overview,” Cell Journal 16, no. 4 (2015): 386–391. 25685729 10.22074/cellj.2015.485 PMC4297477

[35] Y. Lan , H. Tang , Z. Lin , C. Huang , and L. Chen , “Association of Oxidative Balance Score With All‐Cause Mortality Among Individuals With Chronic Kidney Disease: A Cohort Study,” Journal of Health, Population, and Nutrition 43, no. 1 (2024): 160. 39407307 10.1186/s41043-024-00657-6 PMC11481546

[36] J. Li , Y. Liu , J. Li , et al., “Association Between the Oxidative Balance Score With Metabolic Syndrome Traits in US Adults,” Diabetology & Metabolic Syndrome 16, no. 1 (2024): 263. 39497207 10.1186/s13098-024-01500-y PMC11536893

[37] S. Wang , R. Jiang , L. Zhang , Y. Cai , C. Zhou , and L. Wu , “Relationships Between Oxidative Balance Score and Asthma, COPD, With Asthma‐COPD Overlap in American Adults: Findings From NHANES 2013‐2018,” Journal of Asthma: Official Journal of the Association for the Care of Asthma (2024): 1–9, 10.1080/02770903.2024 .2422419. 39453786

[38] Q. Zhang , J. Yi , and Y. Wu , “Oxidative Stress and Inflammation Mediate the Association Between Elevated Oxidative Balance Scores and Improved Sleep Quality: Evidence From NHANES,” Frontiers in Nutrition 11 (2024): 1469779. 39494313 10.3389/fnut.2024.1469779 PMC11528468

[39] F. Hajizadeh‐Sharafabad , Z. Ghoreishi , V. Maleki , and A. Tarighat‐Esfanjani , “Mechanistic Insights Into the Effect of Lutein on Atherosclerosis, Vascular Dysfunction, and Related Risk Factors: A Systematic Review of In Vivo, Ex Vivo and In Vitro Studies,” Pharmacological Research 149 (2019): 104477. 31605782 10.1016/j.phrs.2019.104477

[40] A. Sen , G. Marsche , P. Freudenberger , et al., “Association Between Higher Plasma Lutein, Zeaxanthin, and Vitamin C Concentrations and Longer Telomere Length: Results of the Austrian Stroke Prevention Study,” Journal of the American Geriatrics Society 62, no. 2 (2014): 222–229. 24428184 10.1111/jgs.12644 PMC4234001

[41] N. T. Stringham , M. Green , W. Roche , A. Prado‐Cabrero , R. Mulcahy , and J. Nolan , “Lutein, Zeaxanthin, and Meso‐Zeaxanthin Supplementation Attenuates Inflammatory Cytokines and Markers of Oxidative Cardiovascular Processes in Humans,” Nutrition, Metabolism, and Cardiovascular Diseases 34, no. 8 (2024): 1976–1983. 10.1016/j.numecd.2024.05.009 38890092

[42] F. Zhang , D. Shi , X. Wang , Y. Zhang , W. Duan , and Y. Li , “β‐Cryptoxanthin Alleviates Myocardial Ischaemia/Reperfusion Injury by Inhibiting NF‐κB‐Mediated Inflammatory Signalling in Rats,” Archives of Physiology and Biochemistry 128, no. 4 (2022): 1128–1135. 32362203 10.1080/13813455.2020.1760302

[43] C. H. Hennekens , J. E. Buring , J. E. Manson , et al., “Lack of Effect of Long‐Term Supplementation With Beta Carotene on the Incidence of Malignant Neoplasms and Cardiovascular Disease,” New England Journal of Medicine 334, no. 18 (1996): 1145–1149. 8602179 10.1056/NEJM199605023341801

[44] G. S. Omenn , G. E. Goodman , M. D. Thornquist , et al., “Effects of a Combination of Beta Carotene and Vitamin A on Lung Cancer and Cardiovascular Disease,” New England Journal of Medicine 334, no. 18 (1996): 1150–1155. 8602180 10.1056/NEJM199605023341802

[45] J. M. Rapola , J. Virtamo , S. Ripatti , et al., “Randomised Trial of Alpha‐Tocopherol and Beta‐Carotene Supplements on Incidence of Major Coronary Events in Men With Previous Myocardial Infarction,” Lancet 349, no. 9067 (1997): 1715–1720. 9193380 10.1016/S0140-6736(97)01234-8

[46] L. Schwingshackl , H. Boeing , M. Stelmach‐Mardas , et al., “Dietary Supplements and Risk of Cause‐Specific Death, Cardiovascular Disease, and Cancer: A Systematic Review and Meta‐Analysis of Primary Prevention Trials,” Advances in Nutrition 8, no. 1 (2017): 27–39. 28096125 10.3945/an.116.013516 PMC5227980

[47] G. M. Lowe , L. A. Booth , A. J. Young , and R. F. Bilton , “Lycopene and Beta‐Carotene Protect Against Oxidative Damage in HT29 Cells at Low Concentrations but Rapidly Lose This Capacity at Higher Doses,” Free Radical Research 30, no. 2 (1999): 141–151. 10193582 10.1080/10715769900300151

[48] M. M. Ciccone , F. Cortese , M. Gesualdo , et al., “Dietary Intake of Carotenoids and Their Antioxidant and Anti‐Inflammatory Effects in Cardiovascular Care,” Mediators of Inflammation 2013 (2013): 782137. 24489447 10.1155/2013/782137 PMC3893834

[49] P. Borel , “Genetic Variations Involved in Interindividual Variability in Carotenoid Status,” Molecular Nutrition & Food Research 56, no. 2 (2012): 228–240. 21957063 10.1002/mnfr.201100322

