# Supplementary material for: Inverse Relationship Between Serum Carotenoid Levels and Cardiovascular‐Kidney‐Metabolic Syndrome Among the General Adult Population
Source: J Diabetes. 2025 Jan 30;17(2):e70046. doi: 10.1111/1753-0407.70046 (PMC11780278; doi:10.1111/1753-0407.70046)
Supplement: Supplementary file 1 — Data S1. Supporting Information. [file JDB-17-e70046-s001.docx]

**Online Supplementary Material**

**Inverse Relationship Between Serum Carotenoid Levels and Cardiovascular-Kidney-Metabolic Syndrome Among the General Adult Population**

**Figure S1.** Flowchart of the study participants.

**Figure S2.** Weighted quantile sum (WQS) regression analysis of the association between a combination of six serum carotenoids and components of CKM syndrome among adults in NHANES 2017–2018.

**Table S1.** Distributions and concentrations of serum carotenoids and dietary carotenoid intake levels among adults in NHANES 20117–2018.

**Table S2.** Survey-weighted characteristics of adults ≥20 years by CKM stage in the fasting subsample of NHANES 2017–2018.

**Table S3.** ORs (95% CIs) of the prevalence of advanced CKM syndrome (Stages 3 or 4) according to quartiles of serum carotenoids levels with further adjustment of respective dietary carotenoid intakes among adults in NHANES 2017–2018.

**Table S4.** ORs (95% CIs) of the prevalence of advanced CKM syndrome (Stages 3 or 4) according to quartiles of serum carotenoids levels with further adjustment of dietary carotenoid supplements among adults in NHANES 2017–2018.

**Table S5.** ORs (95% CIs) of the prevalence of advanced CKM syndrome (Stages 3 or 4) according to quartiles of serum carotenoids levels with further adjustment of respective dietary carotenoid intakes and carotenoid supplements among adults in NHANES 2017–2018.

**Table S6.** Associations of quartiles of oxidative balance score (OBS) with the prevalence of advanced CKM syndrome (Stages 3 or 4) among adults in NHANES 2017–2018.

**Table S7.** Associations of quartiles of dietary carotenoid intakes with the prevalence of advanced CKM syndrome (Stages 3 or 4) among adults in NHANES 2017–2018.

**Materials and methods**

**Definition of cardiovascular-kidney-metabolic syndrome stages**

Cardiovascular-kidney-metabolic (CKM) syndrome stages were classified using data from NHANES 2017-2018, according to the 2023 AHA Presidential Advisory on CKM Health [1]. Definitions were adapted based on available NHANES data:

- **CKM Stage 0:** Participants with normal body mass index (BMI, <25 kg/m²), waist circumference (<88 cm for women, <102 cm for men), normoglycemia (fasting blood glucose [FBG] <100 mg/dL, glycated hemoglobin [HbA1c] <5.7%), normotension (systolic blood pressure [BP] <130 mmHg, diastolic BP <80 mmHg), normal lipid profile (triglycerides <135 mg/dL), and no evidence of chronic kidney disease (CKD) or clinical/subclinical cardiovascular disease (CVD).
- **CKM Stage 1:** Participants with elevated BMI (≥25 kg/m²), increased waist circumference (≥88 cm for women, ≥102 cm for men), or prediabetes (HbA1c 5.7%-6.4% or FBG 100-125 mg/dL), without the presence of other metabolic risk factors or CKD.
- **CKM Stage 2:** Participants with metabolic risk factors or moderate-to-high-risk CKD per KDIGO guidelines [2]. Metabolic risk factors included elevated triglycerides (≥135 mg/dL), hypertension, diabetes, or metabolic syndrome (≥3 of the following: elevated waist circumference, low HDL [<40 mg/dL for men, <50 mg/dL for women], elevated triglycerides [≥150 mg/dL], elevated BP [systolic ≥130 mmHg, diastolic ≥80 mmHg], or prediabetes).
- **CKM Stage 3:** Participants with very-high-risk CKD (KDIGO criteria) or high 10-year CVD risk (≥20%) based on the AHA PREVENT equations [3]. High risk was defined as ≥20% 10- year CVD risk (based on recommended thresholds [https://professional.heart.org/en/guidelines-and-statements/prevent-calculator]). Very high-risk CKD was characterized by either Stage G4 or G5 CKD (GFR < 30 mL/min/1.73 m²) or a classification of very high risk based on KDIGO guidelines, determined by GFR and urinary albumin-to-creatinine ratio [2].
- **CKM Stage 4:** Participants with self-reported established CVD, including coronary heart disease, angina, myocardial infarction, heart failure, and stroke. Atrial fibrillation and peripheral artery disease were not included due to data unavailability.

**
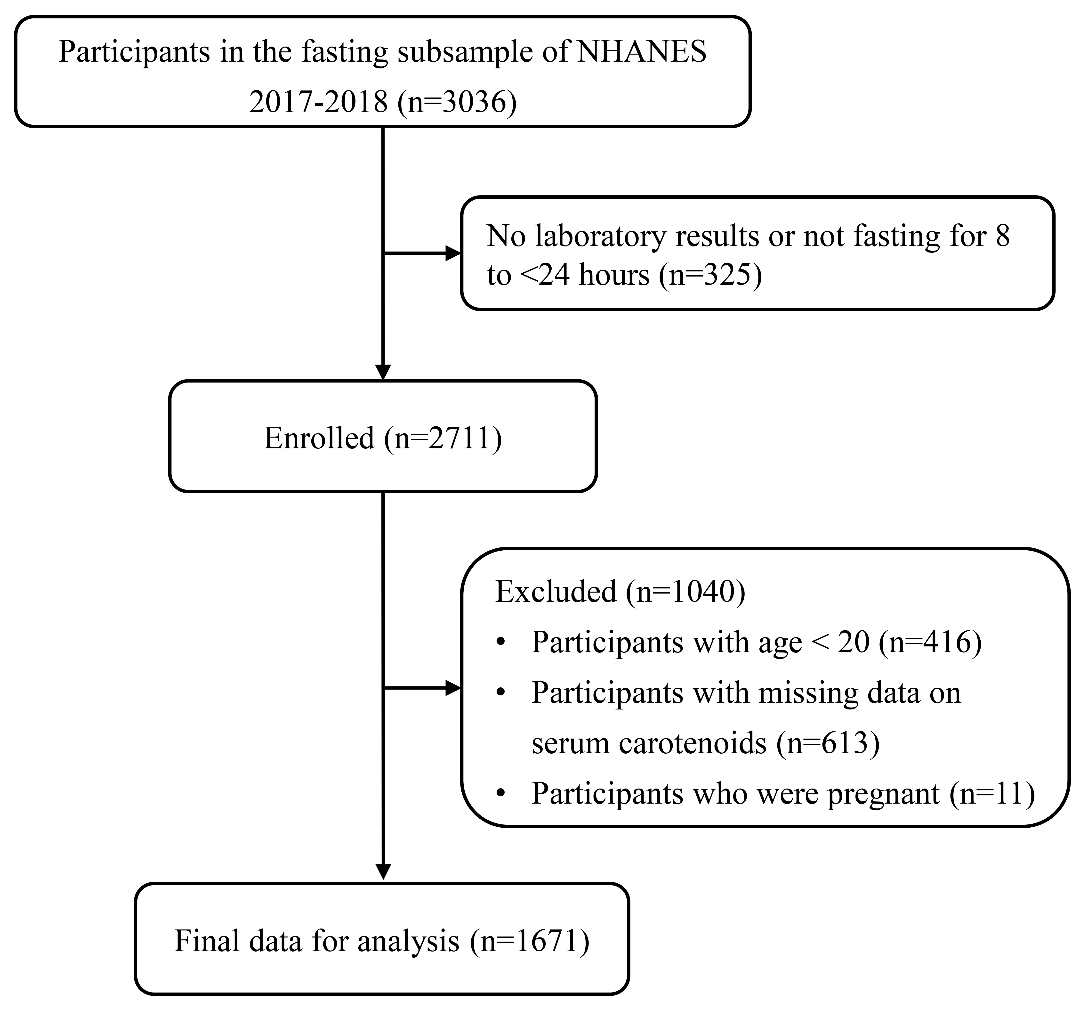
**

**Figure S1.** Flowchart of the study participants

**
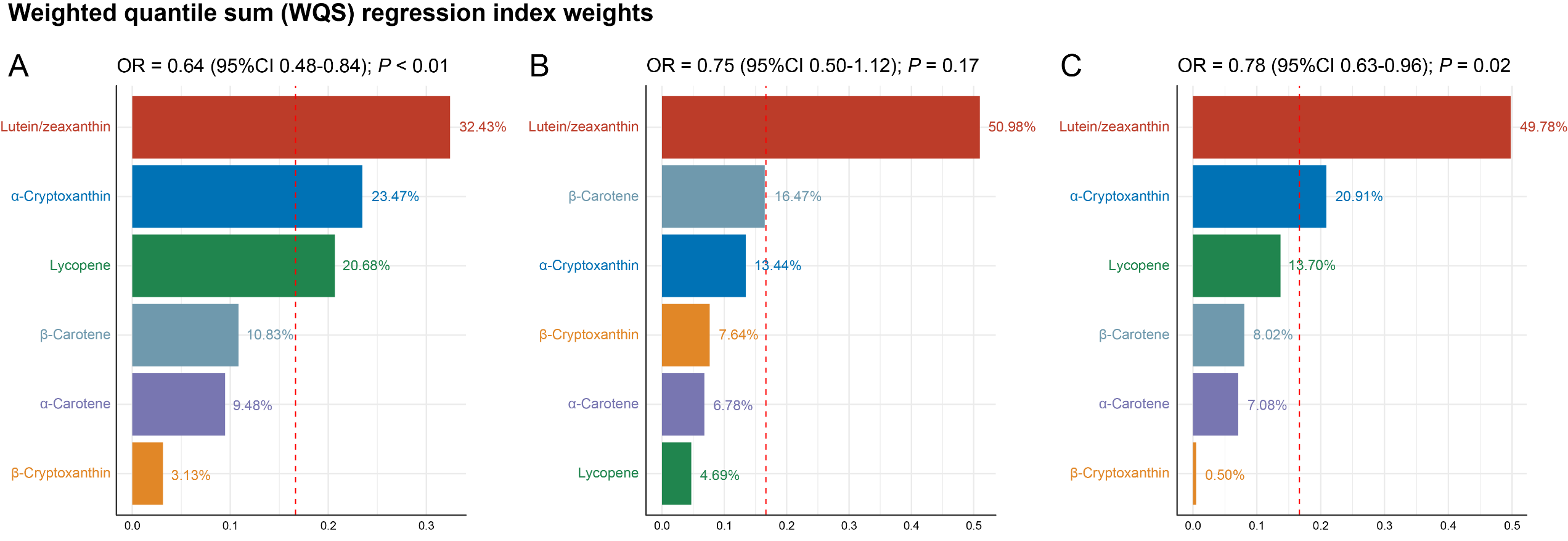
**

**Figure S2.** Weighted quantile sum (WQS) regression analysis of the association between a combination of six serum carotenoids and components of CKM syndrome among adults in NHANES 2017–2018. The analyses were conducted separately for (A) cardiovascular disease (CVD), (B) chronic kidney disease (CKD), and (C) combined CVD and CKD. The WQS regression models were adjusted for age (continuous), sex (male or female), race/ethnicity (Non-Hispanic White, Non-Hispanic Black, or Other), education level (below high school, high school, or above high school), family poverty income ratio (PIR; ≤1.0, 1.1–3.0, or >3.0), serum cotinine (<0.05, 0.05–2.99, or >2.99), drinking status (nondrinker, low-to-moderate drinker, or heavy drinker), physical activity (inactive, insufficiently active, or active), and Healthy Eating Index (HEI; continuous). Abbreviation: WQS, weighted quantile sum regression.

**Table S1.** Distributions and concentrations of serum carotenoids and dietary carotenoid intake levels among adults in NHANES 20117–2018.

|  | Mean | 5^th^ | | 25^th^ | 50^th^ | | 75^th^ | 95^th^ | |
| --- | --- | --- | --- | --- | --- | --- | --- | --- | --- |
| ***Serum carotenoids*** |  | |  | | |  | | |  |
| α-Carotene, μg/dL | 5.33 | 0.50 | | 1.50 | 2.81 | | 5.51 | 17.30 | |
| β-Carotene, μg/dL | 20.42 | 3.76 | | 7.92 | 13.60 | | 23.11 | 60.01 | |
| α-Cryptoxanthin, μg/dL | 2.60 | 0.90 | | 1.63 | 2.28 | | 3.22 | 5.48 | |
| β-Cryptoxanthin, μg/dL | 9.08 | 2.03 | | 3.90 | 6.31 | | 10.50 | 25.20 | |
| Lutein/zeaxanthin, μg/dL | 19.49 | 6.84 | | 11.00 | 16.10 | | 23.30 | 42.00 | |
| Lycopene, μg/dL | 39.83 | 13.60 | | 25.80 | 37.50 | | 50.40 | 77.20 | |
| ***Dietary carotenoids*** |  | |  | | |  | | |  |
| α-Carotene, μg/day | 376.97 | 0.00 | | 11.00 | 45.00 | | 186.00 | 2088.00 | |
| β-Carotene, μg/day | 2412 | 63.00 | | 327.00 | 797.00 | | 2607.00 | 10031.00 | |
| β-Cryptoxanthin, μg/day | 87.97 | 0.00 | | 7.00 | 26.00 | | 80.00 | 368.00 | |
| Lycopene, μg/day | 1613.37 | 84.00 | | 350.00 | 782.00 | | 1551.00 | 5446.00 | |
| Lutein/zeaxanthin, μg/day | 5044.88 | 0.00 | | 1.00 | 1756.00 | | 6213.00 | 21198.00 | |

5^th^, 5th percentile; 25^th^, 25th percentile; 50^th^, 50th percentile; 75^th^, 75th percentile; 95^th^, 95th percentile.

**Table S2.** Survey-weighted characteristics of adults ≥20 years by CKM stage in the fasting subsample of NHANES 2017–2018.

| Characteristics | Stage 0 | Stage 1 | Stage 2 | Stage 3 | Stage 4 | *P* value*** |
| --- | --- | --- | --- | --- | --- | --- |
| Participants, n | 113 (8.5) | 295 (22.5) | 872 (51.7) | 177 (7.1) | 214 (10.3) |  |
| Age, years | 35.01 (1.48) | 38.40 (1.37) | 48.78 (0.98) | 74.74 (0.61) | 64.12 (0.92) | <0.01 |
| Sex, % |  |  |  |  |  | 0.17 |
| Female | 76 (62.14) | 164 (51.74) | 457 (50.54) | 82 (50.16) | 93 (42.02) |  |
| Male | 37 (37.86) | 131 (48.26) | 415 (49.46) | 95 (49.84) | 121 (57.98) |  |
| Race/ethnicity, % |  |  |  |  |  | 0.16 |
| Non-Hispanic White | 47 (71.00) | 84 (60.52) | 247 (59.12) | 85 (70.66) | 95 (66.99) |  |
| Non-Hispanic Black | 18 (7.24) | 77 (12.99) | 205 (11.98) | 38 (10.74) | 59 (13.99) |  |
| Other race | 48 (21.76) | 134 (26.49) | 420 (28.90) | 54 (18.60) | 60 (19.02) |  |
| Education level, % |  |  |  |  |  | 0.02 |
| Below high school | 15 (8.87) | 43 (7.21) | 187 (12.54) | 40 (15.33) | 64 (21.23) |  |
| High school | 24 (21.27) | 51 (21.83) | 214 (32.00) | 54 (33.45) | 48 (29.43) |  |
| Above high school | 74 (69.86) | 201 (70.96) | 471 (55.46) | 83 (51.23) | 102 (49.35) |  |
| Family PIR, % |  |  |  |  |  | 0.03 |
| ≤1.0 | 17 (11.50) | 49 (9.12) | 167 (12.84) | 28 (10.04) | 40 (14.37) |  |
| 1.1–3.0 | 50 (39.75) | 118 (31.63) | 378 (39.13) | 87 (48.40) | 111 (49.12) |  |
| >3.0 | 46 (48.75) | 128 (59.25) | 327 (48.04) | 62 (41.56) | 63 (36.50) |  |
| Serum cotinine, ng/mL |  |  |  |  |  | 0.40 |
| < 0.05 | 61 (56.43) | 167 (60.37) | 476 (55.53) | 122 (72.51) | 111 (53.51) |  |
| 0.05-2.99 | 23 (19.09) | 57 (16.88) | 172 (17.02) | 34 (17.21) | 45 (19.85) |  |
| >2.99 | 29 (24.48) | 71 (22.75) | 224 (27.45) | 21 (10.28) | 58 (26.64) |  |
| Drinking status, % |  |  |  |  |  | <0.01 |
| Nondrinker | 27 (18.03) | 65 (14.76) | 252 (21.40) | 82 (44.93) | 97 (35.58) |  |
| Low-to-moderate drinker | 77 (68.36) | 206 (73.27) | 532 (66.71) | 86 (51.49) | 103 (59.41) |  |
| Heavy drinker | 9 (13.61) | 24 (11.97) | 88 (11.88) | 9 (3.58) | 14 (5.01) |  |
| Physical activity, % |  |  |  |  |  | <0.01 |
| Inactive | 13 (11.58) | 47 (17.76) | 217 (18.98) | 69 (33.18) | 91 (37.47) |  |
| Insufficiently active | 32 (27.33) | 69 (23.89) | 272 (31.51) | 59 (30.90) | 53 (29.39) |  |
| Active | 68 (61.09) | 179 (58.35) | 383 (49.51) | 49 (35.92) | 70 (33.14) |  |
| Healthy eating index | 52.14 (2.88) | 49.97 (1.42) | 48.42 (0.74) | 50.15 (1.14) | 48.30 (2.03) | 0.30 |
| α-Carotene, μg/dL | 3.37  (1.73,9.08) | 3.36  (1.76,7.05) | 2.57  (1.45,4.95) | 2.77  (1.66,4.32) | 2.41  (1.22,3.98) | 0.07 |
| β-Carotene, μg/dL | 18.49  (9.06,33.70) | 15.10  (9.11,28.60) | 11.93  (7.59,19.87) | 15.20  (7.81,23.81) | 12.70  (7.25,18.66) | 0.02 |
| α-Cryptoxanthin, μg/dL | 2.96  (1.94,3.86) | 2.70  (1.90,3.50) | 2.24  (1.62,3.12) | 1.70  (1.21,2.38) | 1.86  (1.23,2.64) | <0.01 |
| β-Cryptoxanthin, μg/dL | 7.90  (4.44,12.20) | 7.72  (4.74,11.50) | 6.17  (3.89,10.10) | 4.50  (2.65, 6.66) | 4.62  (2.79, 9.18) | <0.01 |
| Lutein/zeaxanthin, μg/dL | 17.50  (12.10,22.50) | 17.80  (12.00,25.00) | 15.60  (10.90,23.40) | 15.40  (10.70,20.10) | 15.50  (9.91,20.10) | 0.06 |
| Lycopene, μg/dL | 37.60  (30.80,47.60) | 38.80  (27.10,50.30) | 38.50  (27.50,52.70) | 29.20  (20.20,39.10) | 32.00  (18.20,47.70) | <0.01 |
| Components of CKM Stage | |  |  |  |  |  |
| Body mass index, kg/m² | 22.17(0.14) | 28.61(0.63) | 30.70(0.61) | 30.03(0.54) | 30.44(0.61) | <0.01 |
| Waist circumference, cm | 80.83(0.66) | 96.09(0.98) | 103.36(1.33) | 105.24(0.98) | 105.86(1.64) | <0.01 |
| Systolic BP, mmHg | 108.22(0.68) | 112.81(0.70) | 126.33(0.63) | 143.04(2.76) | 134.60(1.89) | <0.01 |
| Diastolic BP, mmHg | 64.95(0.81) | 69.05(0.56) | 75.99(0.43) | 68.91(1.15) | 72.07(1.73) | <0.01 |
| Fasting blood glucose, mg/dL | 94.0(90.0, 97.0) | 101.0(96.0,107.0) | 105.0(98.0,113.0) | 117.0(105.0,137.0) | 109.0(100.0,133.0) | <0.01 |
| HbA1c, % | 5.10(5.00,5.30) | 5.40(5.10,5.50) | 5.50(5.30,5.90) | 6.10(5.70,6.70) | 5.80(5.50,6.70) | <0.01 |
| Triglycerides, mg/dL | 57.0(45.0, 74.0) | 65.0(51.0, 87.0) | 110.0(72.0,160.0) | 118.0(82.0,154.0) | 120.0(82.0,163.0) | <0.01 |
| HDL-C, mg/dL | 62.0(49.0,72.0) | 56.0(49.0,66.0) | 49.0(41.0,60.0) | 50.0(41.0,61.0) | 47.0(39.0,60.0) | <0.01 |
| eGFR, mL/min/1.73m^2^ | 105.79(96.49,121.37) | 104.58(87.81,118.60) | 97.00(85.24,108.56) | 63.77(51.49, 75.52) | 81.46(64.91, 94.50) | <0.01 |
| Hypertension, % |  |  |  |  |  | <0.01 |
| No | 113(100.00) | 295(100.00) | 197(24.63) | 9(3.97) | 14(7.97) |  |
| Yes | 0(0.00) | 0(0.00) | 675(75.37) | 168(96.03) | 200(92.03) |  |
| Diabetes, % |  |  |  |  |  | <0.01 |
| No | 113(100.00) | 295(100.00) | 668(83.30) | 69(43.74) | 111(57.06) |  |
| Yes | 0(0.00) | 0(0.00) | 204(16.70) | 108(56.26) | 103(42.94) |  |
| Metabolic syndrome, % |  |  |  |  |  | <0.01 |
| No | 113(100.00) | 295(100.00) | 359(41.78) | 48(23.34) | 67(26.83) |  |
| Yes | 0(0.00) | 0(0.00) | 513(58.22) | 129(76.66) | 147(73.17) |  |
| CVD, % | |  |  |  |  | <0.01 |
| No | 113(100.00) | 295(100.00) | 872(100.00) | 177(100.00) | 0(0.00) |  |
| Yes | 0(0.00) | 0(0.00) | 0(0.00) | 0(0.00) | 214(100.00) |  |
| CKD, % | |  |  |  |  | <0.01 |
| No | 113(100.00) | 295(100.00) | 740(84.78) | 80(38.04) | 126(70.68) |  |
| Yes | 0(0.00) | 0(0.00) | 132(15.22) | 97(61.96) | 88(29.32) |  |
| Very high-risk CKD, %† | |  |  |  |  | <0.01 |
| No | 113(100.00) | 295(100.00) | 872(100.00) | 156(91.17) | 195(94.88) |  |
| Yes | 0(0.00) | 0(0.00) | 0(0.00) | 21(8.83) | 19(5.12) |  |
| 10-Year CVD risk, % ‡ |  |  |  |  |  | <0.01 |
| Low-intermediate risk (<20%) | 113(100.00) | 295(100.00) | 872(100.00) | 3(1.05) | 120(59.84) |  |
| High risk (≥20%) | 0(0.00) | 0(0.00) | 0(0.00) | 174(98.95) | 94(40.16) |  |

Abbreviation: PIR, poverty income ratio; CKM, cardiovascular-kidney-metabolic; HDL-C, high-density lipoprotein cholesterol; eGFR, estimated glomerular filtration rate; CVD, cardiovascular disease; CKD, chronic kidney disease. Normally distributed continuous variables are described as means ± SEs, and continuous variables without a normal distribution are presented as medians [interquartile ranges]. Sampling weights were applied for calculation of demographic descriptive statistics; N reflect the study sample while percentages reflect the survey-weighted data.

**P*-values were adjusted for multiple comparisons using the Benjamini-Hochberg method to control the false discovery rate.

†10-year cardiovascular risk was estimated with the AHA Predicting Risk of CVD EVENTs (PREVENT) equations [3]. High risk was defined as ≥20% 10- year CVD risk (based on recommended thresholds [https://professional.heart.org/en/guidelines-and-statements/prevent-calculator]) [3].

‡ Very high-risk CKD was defined as stage G4 or G5 chronic kidney disease (GFR < 30 mL/min/1.73 m²) or a very high risk classification according to KDIGO criteria, based on GFR and urinary albumin-to-creatinine ratio [2].

**Table S3.** ORs (95% CIs) of the prevalence of advanced CKM syndrome (Stages 3 or 4) according to quartiles of serum carotenoids levels with further adjustment of respective dietary carotenoid intakes among adults in NHANES 2017–2018.

|  | Serum carotenoids, μg/dL | | | |  |
| --- | --- | --- | --- | --- | --- |
|  | Quartile 1 | Quartile 2 | Quartile 3 | Quartile 4 | *P* _trend_*** |
| α-Carotene | 1 [Reference] | 0.61 (0.34-1.10) | 0.50 (0.24-1.05) | 0.28 (0.15-0.51) | <0.01 |
| β-Carotene | 1 [Reference] | 0.88 (0.31-2.47) | 0.88 (0.38-2.05) | 0.34 (0.15-0.79) | 0.06 |
| α-Cryptoxanthin | 1 [Reference] | 0.60 (0.37-0.96) | 0.36 (0.21-0.64) | 0.21 (0.10-0.45) | <0.01 |
| β-Cryptoxanthin | 1 [Reference] | 0.60 (0.37-0.99) | 0.43 (0.20-0.92) | 0.46 (0.19-1.11) | 0.06 |
| Lutein/zeaxanthin | 1 [Reference] | 0.51 (0.28-0.94) | 0.73 (0.48-1.12) | 0.26 (0.15-0.46) | <0.01 |
| Lycopene | 1 [Reference] | 0.65 (0.33-1.25) | 0.62 (0.32-1.21) | 0.54 (0.31-0.95) | 0.05 |

Model was adjusted for age (continuous), sex (male or female), race (Non-Hispanic White, Non-Hispanic Black or Other), education level (below high school, high school, or above high school), family PIR (≤1.0, 1.1–3.0, or >3.0), serum cotinine (<0.05, 0.05-2.99, or >2.99), drinking status (nondrinker, low-to-moderate drinker, heavy drinker), physical activity (inactive, insufficiently active, or active), HEI (continuous), and respective dietary carotenoid intakes (in quintiles).

**P*-values were adjusted for multiple comparisons using the Benjamini-Hochberg method to control the false discovery rate.

**Table S4.** ORs (95% CIs) of the prevalence of advanced CKM syndrome (Stages 3 or 4) according to quartiles of serum carotenoids levels with further adjustment of dietary carotenoid supplements among adults in NHANES 2017–2018.

|  | Serum carotenoids, μg/dL | | | |  |
| --- | --- | --- | --- | --- | --- |
|  | Quartile 1 | Quartile 2 | Quartile 3 | Quartile 4 | *P* _trend_*** |
| α-Carotene | 1 [Reference] | 0.64 (0.35-1.16) | 0.54 (0.27-1.10) | 0.29 (0.16-0.54) | <0.01 |
| β-Carotene | 1 [Reference] | 0.87 (0.32-2.37) | 0.84 (0.38-1.84) | 0.32 (0.14-0.74) | 0.04 |
| α-Cryptoxanthin | 1 [Reference] | 0.62 (0.38-1.01) | 0.38 (0.22-0.68) | 0.23 (0.11-0.49) | <0.01 |
| β-Cryptoxanthin | 1 [Reference] | 0.59 (0.36-0.97) | 0.44 (0.21-0.92) | 0.50 (0.22-1.15) | 0.07 |
| Lutein/zeaxanthin | 1 [Reference] | 0.49 (0.26-0.93) | 0.73 (0.48-1.10) | 0.24 (0.13-0.43) | <0.01 |
| Lycopene | 1 [Reference] | 0.68 (0.36-1.29) | 0.68 (0.37-1.22) | 0.58 (0.35-0.98) | 0.07 |

Model was adjusted for age (continuous), sex (male or female), race (Non-Hispanic White, Non-Hispanic Black or Other), education level (below high school, high school, or above high school), family PIR (≤1.0, 1.1–3.0, or >3.0), serum cotinine (<0.05, 0.05-2.99, or >2.99), drinking status (nondrinker, low-to-moderate drinker, heavy drinker), physical activity (inactive, insufficiently active, or active), HEI (continuous), and dietary carotenoid supplements (yes or no).

**P*-values were adjusted for multiple comparisons using the Benjamini-Hochberg method to control the false discovery rate.

**Table S5.** ORs (95% CIs) of the prevalence of advanced CKM syndrome (Stages 3 or 4) according to quartiles of serum carotenoids levels with further adjustment of respective dietary carotenoid intakes and carotenoid supplements among adults in NHANES 2017–2018.

|  | Serum carotenoids, μg/dL | | | |  |
| --- | --- | --- | --- | --- | --- |
|  | Quartile 1 | Quartile 2 | Quartile 3 | Quartile 4 | *P* _trend_*** |
| α-Carotene | 1 [Reference] | 0.61 (0.34-1.11) | 0.50 (0.24-1.05) | 0.28 (0.15-0.50) | <0.01 |
| β-Carotene | 1 [Reference] | 0.89 (0.32-2.50) | 0.84 (0.37-1.93) | 0.31 (0.14-0.73) | 0.04 |
| α-Cryptoxanthin | 1 [Reference] | 0.60 (0.37-0.95) | 0.37 (0.21-0.66) | 0.21 (0.10-0.44) | <0.01 |
| β-Cryptoxanthin | 1 [Reference] | 0.60 (0.37-0.99) | 0.43 (0.20-0.94) | 0.46 (0.19-1.12) | 0.06 |
| Lutein/zeaxanthin | 1 [Reference] | 0.50 (0.27-0.94) | 0.72 (0.47-1.10) | 0.24 (0.14-0.42) | <0.01 |
| Lycopene | 1 [Reference] | 0.65 (0.34-1.26) | 0.62 (0.31-1.22) | 0.54 (0.31-0.95) | 0.05 |

Model was adjusted for age (continuous), sex (male or female), race (Non-Hispanic White, Non-Hispanic Black or Other), education level (below high school, high school, or above high school), family PIR (≤1.0, 1.1–3.0, or >3.0), serum cotinine (<0.05, 0.05-2.99, or >2.99), drinking status (nondrinker, low-to-moderate drinker, heavy drinker), physical activity (inactive, insufficiently active, or active), HEI (continuous), respective dietary carotenoids (in quintiles), and dietary carotenoid supplements (yes or no).

**P*-values were adjusted for multiple comparisons using the Benjamini-Hochberg method to control the false discovery rate.

**Table S6.** Associations of quartiles of oxidative balance score (OBS) with the prevalence of advanced CKM syndrome (Stages 3 or 4) among adults in NHANES 2017–2018 (n=1347) *.

|  | Quartiles of oxidative balance score † | | | |  |
| --- | --- | --- | --- | --- | --- |
|  | <14.0 | 14.0-19.0 | 20.0-24.0 | >25.0 | *P* _trend_ |
| Crude | 1 [Reference] | 0.49 (0.36-0.66) | 0.43 (0.23-0.81) | 0.40 (0.25-0.63) | <0.01 |
| Model 1 | 1 [Reference] | 0.30 (0.14-0.64) | 0.28 (0.11-0.70) | 0.33 (0.17-0.67) | <0.01 |
| Model 2 | 1 [Reference] | 0.36 (0.15-0.90) | 0.35 (0.12-1.05) | 0.44 (0.20-0.99) | 0.02 |

Data are presented as OR (95% CI) unless indicated otherwise; Model 1 was adjusted for age (continuous), sex (male or female), and race (Non-Hispanic White, Non-Hispanic Black or Other); Model 2 was adjusted for Model 1 plus education level (below high school, high school, or above high school), family PIR (≤1.0, 1.1–3.0, or >3.0), and Healthy Eating Index (continuous).

*Participants with missing data for more than two of the 20 OBS components were excluded (n = 324), leaving a total of 1,347 participants included in the final analysis.

†OBS (Oxidative Balance Score) was developed by Zhang et al.[4, 5] and validated extensively. It includes 16 dietary factors (fiber, carotenoids, riboflavin, niacin, vitamin B6, total folate, vitamin B12, vitamin C, vitamin E, calcium, magnesium, zinc, copper, selenium, total fat, and iron) and 4 lifestyle factors (body mass index, physical activity, alcohol consumption, and cotinine). Pro-oxidants (e.g., total fat, iron, BMI, alcohol, and cotinine) were scored inversely, while antioxidants were scored positively. The total score ranges from 0 to 40, and participants were categorized into quartiles (Q1–Q4) based on their OBS scores.

**Table S7.** Associations of quartiles of dietary carotenoid intakes with the prevalence of advanced CKM syndrome (Stages 3 or 4) among adults in NHANES 2017–2018.

|  | Dietary carotenoid intakes, μg/day | | | |  |
| --- | --- | --- | --- | --- | --- |
|  | Quartile 1 | Quartile 2 | Quartile 3 | Quartile 4 | *P* _trend_*** |
| α-Carotene |  |  |  |  |  |
| Crude | 1 [Reference] | 0.76 (0.48-1.22) | 0.62 (0.31-1.22) | 0.99 (0.54-1.83) | 0.86 |
| Model 1 | 1 [Reference] | 0.46 (0.24-0.88) | 0.35 (0.15-0.81) | 0.44 (0.22-0.86) | 0.04 |
| Model 2 | 1 [Reference] | 0.54 (0.29-0.99) | 0.46 (0.21-0.99) | 0.63 (0.34-1.15) | 0.41 |
| β-Carotene |  |  |  |  |  |
| Crude | 1 [Reference] | 0.64 (0.44-0.92) | 0.62 (0.38-1.01) | 0.91 (0.51-1.62) | 0.86 |
| Model 1 | 1 [Reference] | 0.43 (0.24-0.76) | 0.47 (0.24-0.90) | 0.54 (0.27-1.08) | 0.41 |
| Model 2 | 1 [Reference] | 0.60 (0.37-1.00) | 0.67 (0.39-1.16) | 1.00 (0.49-2.04) | 0.86 |
| β-Cryptoxanthin | |  |  |  |  |
| Crude | 1 [Reference] | 0.86 (0.40-1.85) | 0.82 (0.44-1.54) | 1.24 (0.62-2.47) | 0.76 |
| Model 1 | 1 [Reference] | 0.86 (0.40-1.85) | 0.66 (0.32-1.38) | 1.13 (0.41-3.14) | 0.93 |
| Model 2 | 1 [Reference] | 0.92 (0.46-1.85) | 0.71 (0.40-1.28) | 1.46 (0.64-3.35) | 0.71 |
| Lutein/zeaxanthin | |  |  |  |  |
| Crude | 1 [Reference] | 0.72 (0.48-1.09) | 0.83 (0.50-1.37) | 0.64 (0.34-1.21) | 0.41 |
| Model 1 | 1 [Reference] | 0.51 (0.26-0.98) | 0.55 (0.28-1.07) | 0.45 (0.19-1.09) | 0.30 |
| Model 2 | 1 [Reference] | 0.61 (0.35-1.05) | 0.79 (0.46-1.34) | 0.69 (0.30-1.58) | 0.71 |
| Lycopene |  |  |  |  |  |
| Crude | 1 [Reference] | 0.92 (0.58-1.47) | 0.83 (0.51-1.36) | 0.62 (0.37-1.02) | 0.04 |
| Model 1 | 1 [Reference] | 1.22 (0.71-2.10) | 1.15 (0.64-2.07) | 0.80 (0.54-1.20) | 0.41 |
| Model 2 | 1 [Reference] | 1.49 (0.90-2.48) | 1.61 (0.83-3.14) | 1.25 (0.84-1.87) | 0.41 |

Data are presented as OR (95% CI) unless indicated otherwise; Model 1 was adjusted for age (continuous), sex (male or female), and race (Non-Hispanic White, Non-Hispanic Black or Other); Model 2 was adjusted for Model 1 plus education level (below high school, high school, or above high school), family PIR (≤1.0, 1.1–3.0, or >3.0), serum cotinine (<0.05, 0.05-2.99, or >2.99), drinking status (nondrinker, low-to-moderate drinker, heavy drinker), physical activity (inactive, insufficiently active, or active), and Healthy Eating Index (continuous).

**P*-values were adjusted for multiple comparisons using the Benjamini-Hochberg method to control the false discovery rate.

**References**

1. Ndumele CE, Neeland IJ, Tuttle KR, Chow SL, Mathew RO, Khan SS, Coresh J, Baker-Smith CM, Carnethon MR, Després JP *et al*: **A Synopsis of the Evidence for the Science and Clinical Management of Cardiovascular-Kidney-Metabolic (CKM) Syndrome: A Scientific Statement From the American Heart Association**. *Circulation* 2023, **148**(20):1636-1664.

2. **KDIGO 2021 Clinical Practice Guideline for the Management of Glomerular Diseases**. *Kidney international* 2021, **100**(4s):S1-s276.

3. Khan SS, Matsushita K, Sang Y, Ballew SH, Grams ME, Surapaneni A, Blaha MJ, Carson AP, Chang AR, Ciemins E *et al*: **Development and Validation of the American Heart Association's PREVENT Equations**. *Circulation* 2024, **149**(6):430-449.

4. Zhang W, Peng SF, Chen L, Chen HM, Cheng XE, Tang YH: **Association between the Oxidative Balance Score and Telomere Length from the National Health and Nutrition Examination Survey 1999-2002**. *Oxidative medicine and cellular longevity* 2022, **2022**:1345071.

5. Lan Y, Tang H, Lin Z, Huang C, Chen L: **Association of oxidative balance score with all-cause mortality among individuals with chronic kidney disease: a cohort study**. *Journal of health, population, and nutrition* 2024, **43**(1):160.
